# Sixteen Years since the Convention on the Rights of Persons with Disabilities: What Have We Learned since Then?

**DOI:** 10.3390/ijerph191811646

**Published:** 2022-09-15

**Authors:** Andrés Martínez-Medina, Sonia Morales-Calvo, Vicenta Rodríguez-Martín, Víctor Meseguer-Sánchez, Valentín Molina-Moreno

**Affiliations:** 1 Health Sciences PhD Program, Catholic University of Murcia, Campus de los Jerónimos nº135, Guadalupe, 30107 Murcia, Spain; 2 Department of Research and Diagnostic Methods in Education, University of Castilla-La Mancha, 45600 Talavera de la Reina, Spain; 3 Department of Social Work and Social Services, University of Castilla-La Mancha, 45600 Talavera de la Reina, Spain; 4 Social Responsibility, Catholic University of Murcia, 30107 Murcia, Spain; 5 Department of Management, University of Granada, 18071 Granada, Spain

**Keywords:** inclusion, disability, social sustainability, Convention on the Rights of Persons with Disabilities, bibliometrics

## Abstract

Persons with disabilities have historically been subjected to discrimination and exclusion, placing them in dangerous situations of social vulnerability. The Convention on the Rights of Persons with Disabilities, passed on the 13th of December 2006, was an important legislative landmark for valuing the rights of this population group. This study involved a bibliometric analysis of 1024 research articles published in Scopus on the social, workplace, educational, and financial inclusion of persons with disabilities since the Convention. The results show an increase in scientific production, and there is also a great deal of multi-disciplinarity, which has led to important breakthroughs for the all-encompassing inclusion of this section of the population. The most productive journals, authors, institutions, and countries, as well as the international cooperation networks, are presented here. The review concludes by setting out the main themes and trends in the research.

## 1. Introduction

Even though concern for social sustainability is progressively spreading in society, occupying a greater role in political and social agendas and giving rise to better levels of inclusion, disability continues to pose a challenge, because the label continues to be a collective in situations of social vulnerability. One billion people are in a vulnerable state due to disability, which represents 15% of the global population (WHO, 2011). A high proportion of this section of the population lives in developing countries, where the combination of poverty and disability is greater [1,2,3]. The results of the *World Report on Aging and Health* show that the number of persons with disabilities will continue to rise, due to population growth, advances in medicine, and the natural aging process [4]. It is therefore necessary to act decisively on the causes of social marginalization of this group, which comes up against a number of barriers leading to the state of structural exclusion [5]. A direct consequence is that their basic rights are sometimes violated, leading to social, labor, educational, economic, and financial exclusion [6], which prevents them taking their place as full citizens [7,8,9,10,11].

Vulnerability is a reality in access to primary education, where approximately 40 million children with disabilities around the world are not enrolled in school [12]. Thus, efforts are being made to guarantee the provision of inclusive, equitable, and quality education by promoting learning opportunities that include diversity [13]. Some of the difficulties identified in access to education are a lack of economic resources, together with deficits in the training of professionals, requiring curricular adaptation based on a universal design, hindering educational itineraries, and preventing their adequate promotion [14,15,16]. For these reasons, education is often characterized by strong discrimination and stigma [17,18,19].

Once the educational stage is over, there are also situations of vulnerability in access to the labor market [20], characterized by a negative approach to hiring, because there is distrust in performance, which is especially strong in the case of people with intellectual disability [21]. Therefore, there are high levels of unemployment compared with their able-bodied peers [22,23,24], which affects their income level. Palmer et al. (2006) found that, in Scotland, people with disabilities, compared with people without disabilities, are twice as likely to have an income below the poverty line, as well as being twice as likely to have a very low income; thus, they are doubly vulnerable [25].

From a gender perspective, women with disabilities exhibit greater social vulnerability compared with women without disabilities, presenting higher levels of structural gender violence [26]. In India, Mohapatra & Mohanty (2005) found that almost all women and girls with disabilities were subjected to beatings in the home, 25% had been raped, and 6% had been forcibly sterilized [27]. The United Nations states that “*more than half of women with disabilities have suffered physical abuse, while for non-disabled women this figure is one third*” [28].

This discrimination includes the access to and use of financial products and services [29]. In Spain, 17.8% of people with disabilities have suffered financial discrimination in access to insurance: insurance companies often increase prices or include additional clauses, compared with people without disabilities [8]. Beisland & Mersland (2012) reported that 22% of economically active persons with disabilities do not approach financial institutions through fear that their credit application will be rejected merely because of their disability [30].

With the aim of overcoming this situation of multidimensional vulnerability, the Convention on the Rights of Persons with Disabilities (henceforth, Convention), passed on the 13th of December 2006, sets out as a goal: “*promoting, protecting and guaranteeing the full and equal enjoyment of all human rights and basic freedoms by all persons with disabilities, and promoting respect for their inherent dignity*” [31]. Years later, the United Nations declared that States must introduce “*a full and effective inclusion of people with disabilities, and their participation in all dimensions of society*” [32].

Currently, disability is understood as a complex phenomenon that develops as a consequence of the interaction between individual characteristics and the social environment [33]. Consequently, it is necessary to provide strategies and tools to people with disabilities to improve their empowerment, as well as to act on the social environment to achieve a balance that allows the full and effective participation of people with disabilities under equal conditions.

Increased social inclusion of this population group would lead to greater opportunities for community involvement [34,35] and improvements in self-determination [36,37] and decision-making capacity [38]. Social inclusion improves the lives of persons with and without disabilities [39,40], enabling them to contribute to society [41], which is the best weapon for fighting social vulnerability [39].

In this way, the aim of this study was to analyze the scientific knowledge with respect to social, workplace, educational, and financial inclusion (henceforth, social inclusion) of persons with disabilities since the Convention, with the purpose of demonstrating the main research breakthroughs in the field up to the present; the subjects and trends in research; and finally, the gaps in knowledge.

In this field, the authors have found a number of systematic review studies, but no scientometric analyses, and therefore performed a retrospective bibliometric analysis of the first 16 years, looking at the context and approach of research articles published on social inclusion and disability since the Convention. We thus set out five research questions (Q):

**Q1.** What are the main characteristics of the line of research?
**Q2.** Which are the most influential publications?
**Q3.** Who are the most prolific contributors (journals, authors, institutions, and countries)?
**Q4.** What is the trend in collaboration (authors, institutions, and countries)?
**Q5.** Who are the main subjects and what are the research trends?

Consequently, if the social inclusion of people with disabilities improves their levels of empowerment and their health status, our findings would be useful as a basis for future research to delve into the detailed study of some research topics detected in this analysis, thus developing new strategies, tools, and instruments to improve the quality of life of people with disabilities.

Our research is organized as follows: Section 2 describes the methodology used. Section 3 sets out the results obtained for the characteristics of the line of research (Q1) and the most influential publications (Q2). Section 4 presents the contributions and collaborations of journals, authors, institutions, and countries (Q3 and Q4). Section 5 illustrates the main subjects and research trends in social inclusion and disability (Q5). Finally, Section 6 sets out the main conclusions of the retrospective analysis.

## 2. Methods and Materials

### 2.1. Methodology Applied to the Data Analysis

The methodology of this study paper is based on scientometrics or bibliometrics, with the goal of identifying, organizing, extracting, and analyzing research documents in order to examine the change over time of a given area of knowledge [42,43,44]. This methodology is therefore offered as a broad and systematic general description of the literature, in order to trace the development of social inclusion in persons with disabilities, and to set out a route to trending subjects and methodologies [45,46], thus identifying the degree of interest in the subject [47].

The main elements of the interaction between the concepts of social inclusion and disability are identified and analyzed, presenting the metadata and the available trends in the various databases that reflect this specific subject area [44,48]. In addition, network maps have been generated in order to group and process words, using the Voswiever v.1.16.7. software (see [49,50,51], for example).

### 2.2. Procedure of the Bibliometric Analysis

**Figure 1:**
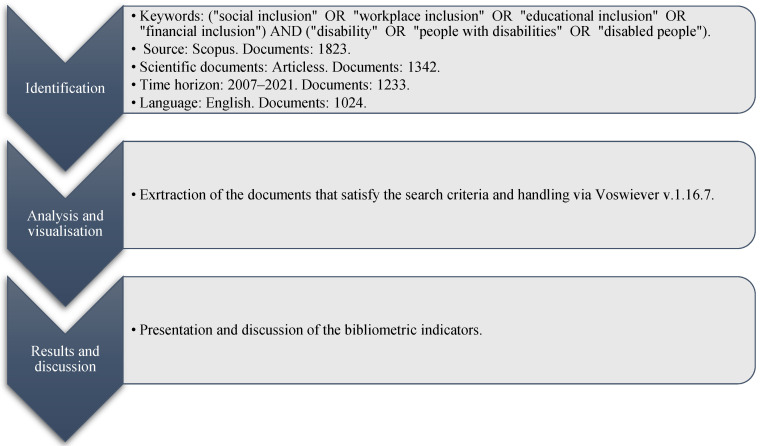
Methodology applied.

The methodology was applied in three stages, as shown in Figure 1.

The methodological procedure used is explained in detail below.

#### 2.2.1. Identification Stage

The main databases consulted were those most closely linked to the field of knowledge, such as Web of Science, Scopus, PubMed, and Google Scholar [52,53]. Scopus was finally chosen because: (a) it is the database with the greatest volume of information about authors, institutions, and countries [54]; (b) it is the repository with the greatest number of articles and reviews that satisfy the quality requirements of scientific peer review [55,56]; and (c) compared with Web of Science, Scopus has greater coverage [57]. Scopus was therefore the most appropriate source for performing this bibliometric analyses [58]; 1823 research documents were found satisfying the search requirements.

The first filter applied was the type of scientific document; only research articles were chosen, because they are assessed based on novelty and are subjected to rigorous peer review, which is an indication of greater scientific quality [59]. For this reason, 481 documents were excluded for not satisfying the search criteria.

Next, a time horizon was applied, for the years immediately after the Convention. This was enacted at the end of 2006; therefore, the period 2007–2021 was selected. As a result, the number of documents that met the search requirements was reduced to 1233.

Finally, only documents that were written in English were chosen, following the recommendations of Donthu et al. (2021), who suggested that performing translations was not practical for reviewing large datasets [58]. Thus, the definitive number satisfying the search requirements was 1024. 

#### 2.2.2. Analysis and Visualization Stage

The data were downloaded and analyzed in January 2022. From the sample of articles satisfying the search requirements, the interactions between authors, countries, institutions, and the development of key words, were analyzed. International cooperative networks enable novel, high-impact research to be produced, and they contribute to the production of synergies and the exchange of ideas [60]. This analysis was caried out by co-citation: As the frequency increased, the inter-relations between them also increased, making for a greater conceptual relationship. 

Keyword analysis, on the other hand, is based on the co-occurrence method, developed to identify a conceptual and thematic structure, such that the results show a general overview of the most widely researched areas in the relationship between craftsmanship and sustainability.

#### 2.2.3. Results and Discussion Stage

The results are shown for authors, journals, subject areas, countries and affiliation, and international cooperation networks, as well as keywords, producing maps based on the co-occurrence of keywords and co-citations, which are widely used in bibliometric studies (see [61,62,63], for example). 

## 3. Main Characteristics of Scientific Production and the Most Influential Publications (Q1 and Q3)

This section presents the main characteristics of scientific production on the line of research of social inclusion and disability since 2007 (inclusive). Then, results are shown for the increasing number of articles published, the number of citations, the researchers, and the countries that have been studying this line of research, as well as the main subject areas. Furthermore, the results are shown for the most influential publications, based on overall citations, i.e., the number of citations for each research article without filtering [64].

Table 1 shows the main characteristics of the scientific production on inclusion and disability. The time horizon was 16 years; therefore, the characteristics were divided into five 3-year periods to facilitate analysis and interpretation.

The number of research articles increased by 1500%, the authors increased by 1700%, the number of contributing countries increased by 1010%, citations increased by 16,625%, and the average number of citations increased by 920%. The average number of co-authors also increased from 2.6 in the first 3-year period (2007–2009) to 3.2 in the last 3 years (2019–2021).

**Figure 2:**
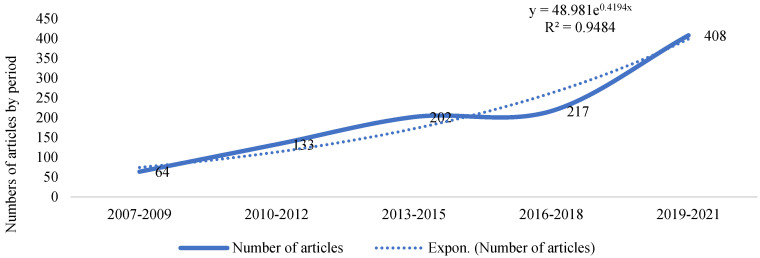
Development of the number of research articles published by 3-year period.

Figure 2 shows the development over time of the number of articles, where an exponential variation between each 3-year period studied can be observed.

The first article published in the line of research was that by Kaiser et al. (1985), who analyzed the use of clothing as a means of “hiding” disability [65]. It is significant that, over the more than two decades prior to the Convention, 108 articles were published analyzing the social inclusion of this group. Sixteen years later, scientific production increased by 848%.

**Figure 3:**
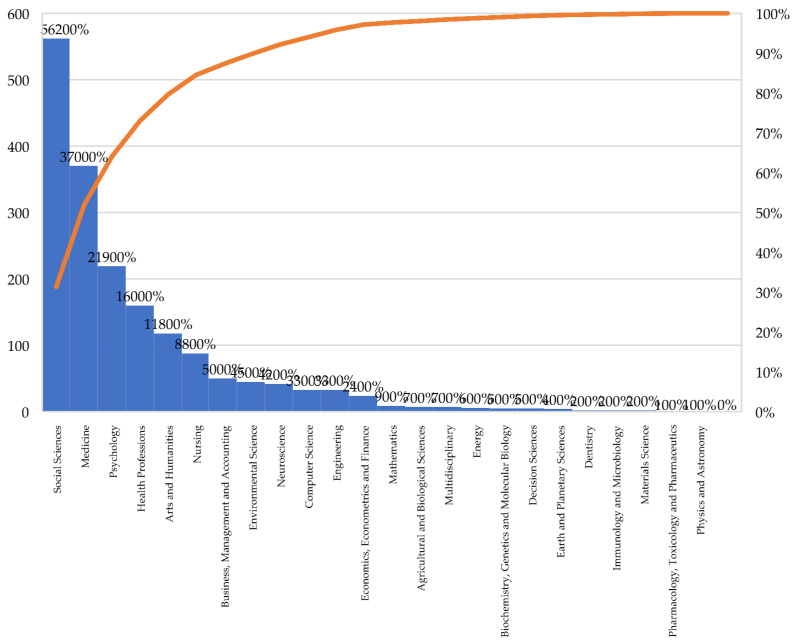
Scientific production by subject areas.

Figure 3 shows the results of the analysis of the subject areas. The 1024 research articles are organized into 24 subject areas, highlighting the multi-disciplinarity in proposing new methodologies and instruments for the real inclusion of people with disabilities.

Thus, Social Science is the subject area that has received the most attention (*n* = 652; 31.31%), followed by Medicine (*n* = 370; 20.61%), Psychology (*n* = 219; 12.20%), Health Professions (*n* = 370; 20.61%) = 160; 8.61%, and Arts and Humanities (*n* = 118; 6.57%).

Growth is observed in all the indicators in the line of research, which indicates that the Convention has progressively encouraged multidisciplinary researchers to analyze the different dimensions of society in which the group would have to be included, thus building an egalitarian, inclusive society, which created opportunities, under equal conditions, for all social actors.

Table 2 presents the 10 most influential research articles.

Solish et al. (2010) analyzed the participation of children with and without disabilities from 5 to 17 years of age in social activities to generate friendships, concluding that the latter participate in a greater number of social activities, for which they consider it important to measure not only the number, but with whom the interactions take place [66]. Along the same lines, Dingle et al. (2013) studied the participation of adults with disabilities in a local choir as a response to “emotional flattening and emotional isolation”, finding that their participation contributed to the development of a social identity [67]. Milner & Kelly (2009) identified five qualitative attributes that people with disabilities develop in their sense of belonging to the community [68]. In their study, Amado et al. (2013) carried out a review on the social participation of people with disabilities, identifying emerging research problems and new areas where research is needed [69].

Hall (2010) studied workplace inclusion and independent living, concluding that the inclusion of people with disabilities is not only an individual benefit, but is also an instrument for the social and cultural understanding of people with disabilities [70], whereas Arvanitis et al. (2009) analyzed the benefits of augmented reality as a tool for educational inclusion, identifying problems and challenges in technological usability and acceptance [71]. Some years later, Bossaert et al. (2013) assessed the concepts of social integration, social inclusion, and social participation for the educational inclusion of this section of the population [72].

McConachie et al. (2015) performed a systematic review of tools for measuring progress and results in ASD children [73], whereas Davidson (2008) previously identified different autistic styles of communication [74]. Finally, O’Brien et al. (2008) designed a conceptual framework for disability in adults with HIV [75]. 

## 4. Contributions and International Cooperation Networks (Q3 and Q4)

This section presents the results of the productivity of the authors, institutions, countries, and journals, as well as their international cooperation networks.

Table 3 shows the 10 most productive authors. The Australians Bigby, C. and Wilson, N.J. are the most prolific authors (16 and 12 published research articles, respectively). Bayoumi, A.M., had the highest average number of citations per article (26.71).

Notably, 50% of authors are of North American origin, followed by 30% of Oceanic origin, and only 20% being Europeans. This shows important co-authorships. The Canadians O’Brien, K.K.; Solomon, P.; and Bayoumi, A.M., work together, with eight co-authored research articles published, similarly to the three Australian authors. For their part, the Canadians Lindsay, S., and McPherson, A.C., co-authored two articles, whereas the European authors have not published jointly, at least in this line of research.

**Figure 4:**
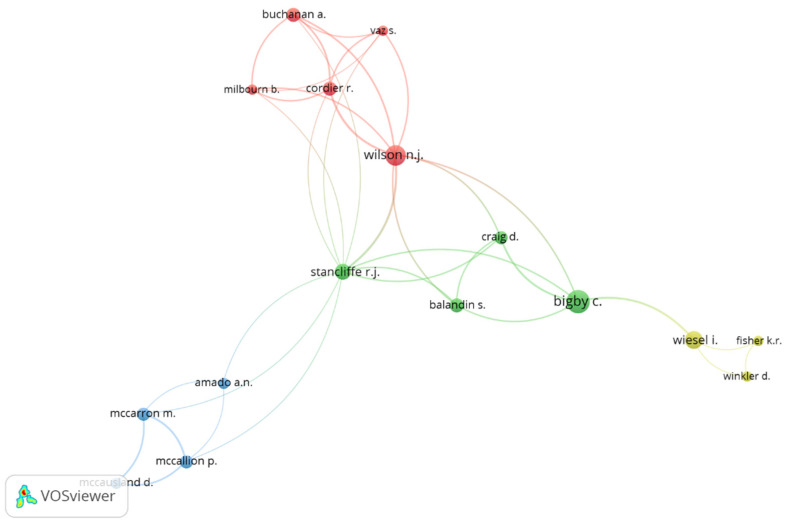
International cooperation networks of the authors on social inclusion and disability.

Figure 4 shows the international cooperation networks of researchers of social inclusion and disability. The colors show the clusters; the sizes of the circles show the volumes of the scientific literature. By selecting an interaction of at least three research articles, 80 co-authors were determined. However, constraining them to work in co-authorship reduced them to a total of 16, grouped into four clusters.

The largest cluster is in red, made up of five co-authors, of whom Wilson, N.J., acts as a connector with the green cluster, the second largest with four co-authors. From there, Sancliffer, R.J., connects the green with the blue cluster, also comprising four co-authors, and Bigby, C., connects the yellow cluster, with three co-authors.

Table 4 shows the 10 most productive institutions. The University of Toronto is the most productive institution (42 published research articles) and has the highest H index (14), followed by the Australian La Trobe University and The University of Sydney (25 research articles each). The University of Toronto also achieved the greatest dissemination of its research results, with 617 total citations, followed by Queen’s University which, despite being the least productive institution (13 publications), achieved 534 total citations, and an average of 41.08, giving it the highest average of citations within the Top 10 most productive institutions.

A high concentration of nationalities stands out, with 50% Australian institutions, 40% Canadian, and the remaining 10% being Irish. La Trobe University, UNSW Sydney, and Monash University frequently publish jointly (four co-authored research articles), and rarely with The University of Sydney (2), whereas the University of Melbourne only published two co-authored research articles. The most notable co-authorship is that of the University of Toronto and Holland Bloorview Kids Rehabilitation, with 16 co-authored publications in this line of research.

With respect to international cooperation networks, it should be noted that the trend is towards reduced international co-authorship because, with the exception of Trinity College Dublin (CI = 96%), the remaining institutions in the Top 10 of the most productive have cooperation rates below 40%, especially Holland Bloorview Kids Rehabilitation Hospital, whose published research articles on inclusion and disability were with national co-authors.

**Figure 5:**
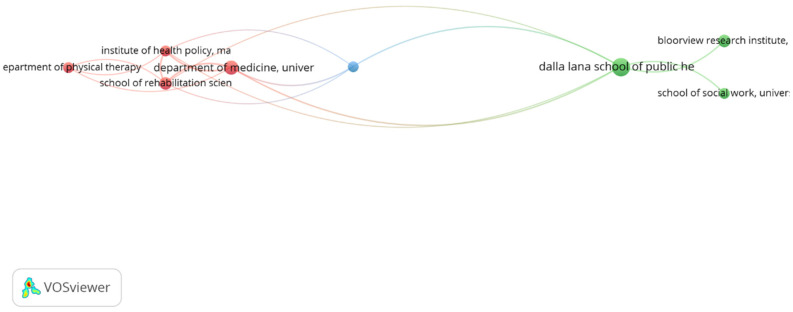
Cooperation networks of institutions in social inclusion and disability.

Selecting an interaction of at least three research articles, eight institutions in total were found, grouped into three clusters (Figure 5). The largest cluster is red, comprising four institutions, three of them from the University of Toronto and the School of Rehabilitation Science, from McMaster University, also a Canadian-based institution. The Department of Medicine of the University of Toronto is affiliated with the Department of Psychiatry, also of the University of Toronto, and which only involves the blue cluster, given its limited international collaboration. It is also, however, associated with the Dalla Lana School of Public Health at the University of Toronto which, together with three other Canadian institutions, constitute the green cluster.

Table 5 shows the 10 most productive countries in the field of social inclusion and disability research over the period 2007–2021.

With respect to origin, unlike authors and institutions, the case of countries is more diversified. In this way, 50% are of European origin, 20% are of North American origin, and the remaining 30% are distributed across Africa, Latin America, and Oceania.

The country with the highest volume of research articles published in the line of research (214) is the United Kingdom, followed by Australia (160); these two countries also have the highest number of total citations (2800 and 1997, respectively) and H index (53). However, The Netherlands is the country with the highest average number of citations (17.47), followed by Canada (14.02) and Ireland (13.69), despite the latter being well below the number of publications in the top three countries in terms of volume.

The tendency to international cooperation is low, and only Australia (70.6%), Ireland (61.1%), and South Africa (55.3%) have cooperation rates above 50%. In fact, except for these three countries and the United Kingdom, the number of international collaborators in each country is very small.

**Figure 6:**
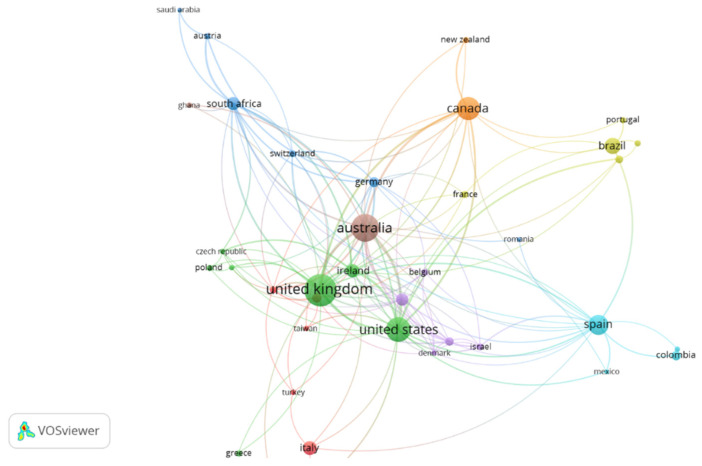
International cooperation networks for the countries in social inclusion and disability.

Figure 6 shows the international cooperation networks for these countries. Selecting an interaction of at least five research articles, 38 countries were found, grouped into eight clusters. 

The largest is the red cluster, comprising seven countries, led by Italy. The green cluster also has seven countries, led by the United Kingdom and the United States, which are also in the center of the figure, indicating that they cooperate with a large number of other countries.

This is followed by the blue cluster, comprising six countries, led by South Africa; the yellow cluster, led by Brazil and made up of five countries; the purple cluster is also made up of five countries and is led by The Netherlands. Spain leads the light blue cluster, made up of four countries. Finally, Canada leads the orange cluster and Australia leads the brown cluster, each comprising only two countries.

Finally, Table 6 shows the Top 10 most productive journals in research on social inclusion and disability: 100% are of European origin, and nationally, 80% are from the United Kingdom.

*Disability and Society* is the most productive journal (44), followed at some distance by *Disability and Rehabilitation* (29) and the *Journal of Applied Research in Intellectual Disabilities* (27). *Journal of Applied Research in Intellectual Disabilities and Disability* and *Society* have the highest volume of total citations (with 712 and 707, respectively), although they are closely followed by the *Journal of Intellectual Disability Research* (692) which, despite its reduced volume of publications compared with the previous two (19), reached the highest average number of citations (36.42).

The *International Journal of Environmental Research and Public Health*, *Disability and Rehabilitation,* and the *Journal of Intellectual Disability Research* have the highest H index values (113, 111, and 104, respectively), although it should be noted that up to 80% of the Top 10 most productive journals are in quartile 1 (Q1) of the SCImago Journal & Country Rank (SJR). 

## 5. Research Topics and Trends in Social Inclusion and Disability (Q5)

**Figure 7:**
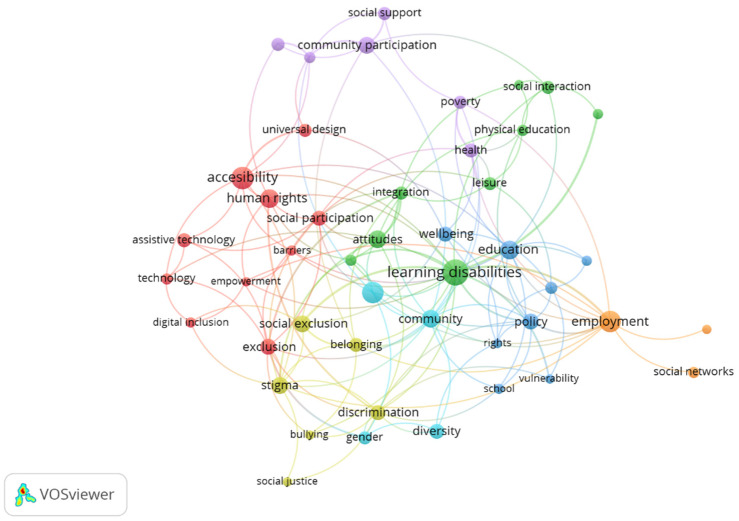
Network of keywords in social inclusion and disability.

Figure 7 shows the relationships of the keywords in the research line of social inclusion and disability in the period 2007–2021. For a total of 2355 keywords contained in the 1024 articles analyzed, a minimum interaction of six co-occurrences was used, giving a final total of 90 keywords. Subsequently, filtering was performed, eliminating those keywords that were incorporated into the search criteria and others that were not related to our study, thus avoiding the possibility of obtaining erroneous or non-representative results. In this way, the number of keywords finally analyzed was 58. The size of each bubble represents the number of times each keyword was repeated in the sample, whereas the lines that join them show which words usually co-appeared in the articles.

Consequently, the keywords were organized around seven clusters, considering the most significant research topics in social inclusion and disability.

**Figure 8:**
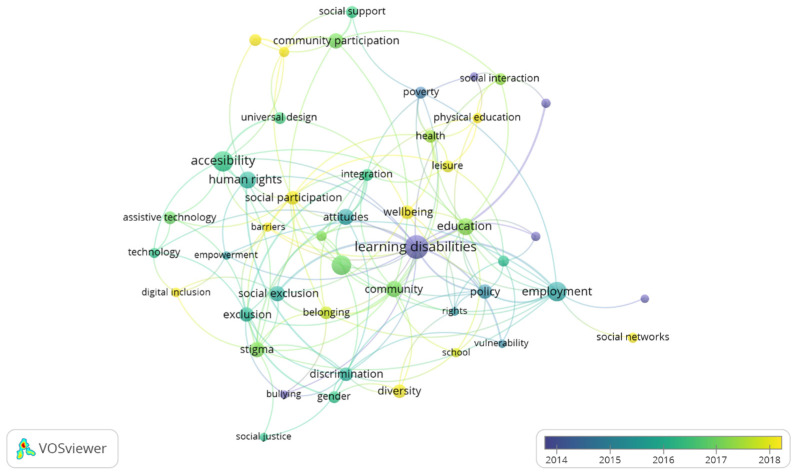
Research trends in social inclusion and disability.

Figure 8 shows the main research trends. The lighter colors show the most recent keywords, indicating a high volume of keywords within each cluster that are becoming very important in recent years. Consequently, we observe that all research topics in social inclusion and disability are being addressed, which implies that the line of research will be providing multidisciplinary solutions in the coming years.

Next, the most important contributions within each topic identified in Figure 7 are described, referencing them to the trends identified in Figure 8.

1. Digital accessibility

The cluster with the most keywords is red, with 10, present in 157 research articles (15% of the sample), referring to digital accessibility.

Cognitive accessibility, through technology, is a key element for the social inclusion of people with intellectual disabilities [71], improving the confidence, safety, protection, and independence of people with disabilities [76], as well as for the generation of interpersonal relationships [77].

Davidson (2008) found that the internet, given its socio-spatial distance, has the potential to contribute to the social inclusion of people on the autism spectrum (AS) [74]. However, Darcy et al. (2017) proposed a personalized use of technology, because not all people with intellectual disabilities acquire and develop learning in the same way, sometimes leading to opposite results [77].

Within the framework of the construction of smart cities, Ramírez et al. (2017) used the Internet of Things (IoT) to help people with intellectual disabilities travel [78], whereas De Oliveira et al. (2016) offer contributions in augmented reality to promote the internal mobility of people with physical disabilities [79]. In the context of socio-health, Hers & Johnson (2008) proposed the Comprehensive Assistive Technology (CAT) model to promote dialogue between people with disabilities, social services, and clinical rehabilitation services, in order to eliminate barriers to full participation [80].

However, digital accessibility constitutes a conceptual framework in development, which implies that the results must be measured individually in each case, because there are still barriers to be overcome [81].

2. Community participation

The green cluster comprises nine keywords present in 153 research articles (15% of the sample) and refers to the community participation of people with disabilities.

Research in this field has analyzed the structural characteristics of social communities of people with disabilities, such as artistic spaces [70,82] and inclusive sports activities [83,84]. It is found that participation in community activities contributes to the development of feelings of attachment and belonging, as well as higher levels of socialization and the creation of social networks [67,85].

However, beyond these structural characteristics, the analysis of the functional characteristics showed that social networks are mainly limited to family members and professionals [86]; thus, it is still necessary to study the development of new mechanisms more deeply, to improve the breadth of social networks of people with disabilities and their community participation.

These barriers aside, Salmon (2013) found that self-exclusion is a valid strategy for generating friendly relationships [87], although for Jaeger & Xie, (2009) the key could be in online interactions [88]. On the other hand, for Giesbers et al. (2019), it is the natural support figures that play a more important role to facilitate and maintain close social relationships between people with intellectual disabilities [89].

3. Education and independent living

The dark blue cluster, which refers to the research topics of education and independent living, comprises eight keywords, which are included in 210 research articles (20.5% of the sample).

In education, important methodological advances have been made in improving the academic results of people with disabilities. Technologically, Arvanitis et al. (2009) proposed an augmented reality model to improve the visual perception of people with physical disabilities [71], whereas Leo et al. (2017) analyzed the influence of smartphones in improving the educational results of people with intellectual disabilities [90].

In the structural aspect, the designs of educational networks [91] or special units [92] are responding to the educational inclusion of people with intellectual disabilities, while learning communities are addressing this issue for people with physical disabilities [93].

In the university setting, Moriña et al. (2017) and Rodríguez et al. (2020) found that providing for people with disabilities requires training academic staff, improvements in the accessibility of facilities, and the development of inclusive higher education programs [94,95].

However, there are still challenges in achieving the educational inclusion of people with disabilities, such as the clarity and poor implementation of educational policies, the ambiguity of the objectives of inclusion, and the means of achieving it [96].

Regarding the independent lives of people with disabilities, this new model of social assistance emerged in the 1970s, and in 2004 it experienced a strong boost in the European Community [97]. Today, we know that it was a success, because their social development in a situation of independence is contributing to the creation of social relationships [89].

However, this new framework for action has also presented some barriers or operational difficulties, such as problems of support, health, choice, and control [98,99]. Figures of natural support represent key elements in reducing these barriers and, therefore, vulnerability, thus achieving higher levels of community inclusion compared with other residential programs, because those who work on these resource designs and perform individualized intervention plans adjust to the needs of each individual with a disability [100].

4. Work

The orange cluster, made up of three keywords, present in 63 research articles (6.15% of the sample), refers to workplace inclusion, and is the topic that has generated the least interest of all those analyzed. This is true despite not only providing people with disabilities with a stable source of income, but also favoring their social inclusion, the creation of broader social networks, greater confidence, and the development of new functional skills [101]. However, people with disabilities often have difficulties in finding a job, with parents and their closest social relationships being the most commonly used resource [102].

Once in a company, acceptance by able-bodied peers depends on sharing common work goals, whether the employer supports equality in the workplace, and whether they come to know their peers as individuals and not as labels [103]. Therefore, it is essential to develop a culture of workplace inclusion within organizations [104]. In this context, tools have been found that contribute to job empowerment: natural support [105], intergenerational mentoring [106], job adaptation [107], and supported employment [108] contribute to equity, self-worth, and sense of belonging. Some studies have been conducted which show the positive impact of the inclusion of people with disabilities in the workplace, which also contributes to promoting the corporate social responsibility of companies, improving their reputation with internal and external stakeholders, and thus being labor-inclusive as a double benefit, with a high social and reputational impact [109].

5. Barriers to social inclusion

The yellow cluster refers to the barriers to social inclusion of people with disabilities, and comprises six keywords, present in 93 research articles (9% of the sample).

Stigmas constitute a barrier to social inclusion [110], causing exclusion and segregation, especially in the case of people with intellectual disabilities [111]. These are present in all the dimensions in which people with disabilities should be included.

In the educational field, Lindsay & McPherson (2012) concluded that the attitude of teachers of children with disabilities often influenced social exclusion by the rest of their classmates [112]. On the other hand, in the technological dimension, Darcy et al. (2017) found resistance in the attitudes of telephone service providers to providing services to people with disabilities [112]. In the field of health, Pelleboer-Gunnink et al. (2017) found that stigmatization by health professionals of people with intellectual disabilities caused them stress, lack of confidence, fear, and anxiety [113].

What is most worrying is that it is not yet clear how to overcome these barriers. For Ouellette-Kuntz et al. (2010), the generation of stigmas is associated with factors such as age and educational level [114]. Rillotta & Nettelbeck (2007) found that information constitutes long-term benefits for developing an inclusive society [115], although McManus et al. (2011) suggested that greater knowledge in society about people with disabilities does not improve attitudes towards them [116].

6. Social support and quality of life

Finally, the purple and light blue clusters refer to social support and quality of life, although these were analyzed as a single cluster given the intersection with all previously identified clusters. This aspect is thus made up of 10 keywords present in 241 research articles (31.5% of the sample).

The situation of historical vulnerability of people with disabilities has given rise to numerous situations of inequality compared with their peers without disabilities [117]. The situation of exclusion is, at the same time, a key determinant of health [118,119], causing worse physical and mental health outcomes at the individual and community levels [120,121,122] and high mortality rates [123,124]. Therefore, many have sought alternative spaces and activities to seek inclusion [70]. However, in the previous clusters, we found that numerous practices and methodologies currently exist, and are achieving positive results, which could contribute to the gradual reduction in the vulnerability of people with disabilities.

All this should contribute to improving the quality of life of people with disabilities. For this reason, in this period, ideas for measuring it have been introduced: Huxley et al. (2012) proposed the Social and Community Opportunities Profile (SCOPE), as a multidimensional index of the social inclusion of people with disabilities [125]; shortly afterwards, Gomez et al. (2015) proposed the INICO-FEAPS scale [126]. However, it is important to be careful when using these metrics, because they may not be representative of the entire population with disabilities [127]. 

In addition to the practices of private institutions, government commitment to developing an equal and inclusive society is also important, because according to MacLachlan et al. (2012) and Mannah et al. (2012), social services in some countries are often not equitable, accessible, or inclusive [128,129]. This leads to people with disabilities facing additional costs involving supplementary expenses when purchasing general products and services, or those directly related to the disability [8], which means that, in England, their minimum income requirements are 50% higher than the state allowance they receive [130]. This, without a doubt, continues to limit their full and equal participation in access to basic social services of quality.

However, the new trends in the management of public social resources are observable, favoring more efficient management aimed at inclusion. In this sense, Australian public administrations allocate resources based on individual needs for people with disabilities, rather than the traditional block funds [131]. Since the Convention, most governments have progressively decided to spend less money on social assistance instead of protecting human rights, leading to a redirection of funds from institutional services to community services in order to overcome this barrier [132].

## 6. Conclusions

The Convention on the Rights of Persons with Disabilities, passed on 13 December 2006, represented a new international political–social context, highlighting the rights of a group which, from that moment, had an equal access framework. This study analyzed the scientific literature on social inclusion and disability since the Convention, carrying out a bibliometric analysis of 1024 research articles available in the Scopus database. There are five major conclusions (Cs):

**C1.** The main characteristics of this line of research show that there has been a considerable growth in scientific production, and therefore, in interest in the inclusion of people with disabilities, after the approval of the Convention. Additionally, considerable multi-disciplinarity is observed, generating proposals for inclusion in all the fundamental rights referred to in the Convention.

**C2.** The most prolific in this line of research have been: Bigby, C., as the most prolific scholar; the University of Toronto, as the most prolific institution; the United Kingdom, as the most prolific country; and *Disability and Society*, as the journal which has published the most articles.

**C3.** International collaboration between authors, institutions, and countries is limited.

**C4.** The main research topics on inclusion and disability are “digital accessibility”, “community participation”, “education and independent living”, “work”, “barriers to social inclusion”, and “social support and quality of life”. In all of these, there are multiple trending keywords, which implies a generalized interest in this line of research. In all of them, natural support figures take on a special importance, and this means any person who, without the need for any specific knowledge, but rather by adopting facilitating, normalized, and empathic skills and attitudes, facilitates the social inclusion of people with disabilities in a normalized way.

However, the authors have found that there are research gaps to be covered, in order to achieve real inclusion and the equal participation of people with disabilities.

These include analyses of the barriers and attitudes to achieve the real inclusion of people with disabilities. Therefore, we propose a line of research focused on the design of a trans-diagnostic assessment model that evaluates individual characteristics and the demands of the environment, to achieve personal objectives. This would not start from the type of disability, but would rather identify a series of variables typical of the individual and the environment, thus determining their degree of individual vulnerability based on the demands of the environment. This, in line with studies that have analyzed figures of natural support, would make it possible to design a personalized intervention system and increase the chances of success in achieving the individual goals of each person with a disability. 

On the other hand, one of our objectives was to analyze the degree of development regarding the financial inclusion of people with disabilities, who were not identified among the topics of inclusion and disability exhibited in Figure 7. The studies in this regard are very scarce and are mainly focused on barriers to access [133,134]. Only Gálvez-Sánchez (2021) has thus far developed a methodological proposal for the financial inclusion of people with disabilities [135]. Therefore, to comply with the guidelines of the Convention, the authors believe that new instruments must be designed for the offer, access, and use of financial products and services adapted to the needs of this section of the population [30].

This study is unique in the literature, setting out as it does the main authors, countries, institutions, and international cooperation networks, as well as the main topics and research trends, and showing the impact of the Convention on the Rights of Persons with Disabilities in the development of a model of an inclusive and egalitarian society. Our results could be valuable for professionals and organizations that care for people with disabilities, who could identify and implement new practices to contribute to their social, educational, and work development. At the same time, they could also be useful for formulators of social policies, who have a scientific basis on which to develop new socio-political measures for the inclusion of people with disabilities in several areas of law. Additionally, given the importance of natural support figures, our results could be valuable for any reader interested in persons with disabilities, finding motivation to adapt their habits and social behaviors and contribute individually or collectively to their social inclusion.

This study raises some limitations that offer suggestions for future research in the area of knowledge. For example, it was based solely on research articles from the Scopus database. In the future, the use of other databases such as Google Scholar or Web of Science, among others, as well as a greater diversity of research documents, such as book chapters or papers presented at conferences, could complement the information obtained here. For its part, the research included the most common and general terms on the subject, which possibly excludes studies that have used more specific terms. Likewise, the search criteria led to the exclusion of documents that could have been highly valuable in the development of this area of research; thus, in the future, expanding the types of documents or their language would enable an analysis much more exhaustive. The computer tool used for visualizing the cluster data was VOSViewer, meaning that the use of other computer software could also provide slightly different results. Finally, the methodology used for the bibliometric analysis does not consider that the citations require time to be analyzed. In the future, content analysis could be complementary, in order to assess the quality of the research.

## Figures and Tables

**Table 1:** Main characteristics of scientific production.

Year	Articles (A)	Authors (AU)	Countries (C)	Citations (TC)	Average Citations (TC/A)
2007–2009	64	164	21	59	0.92
2010–2012	133	332	37	449	3.38
2013–2015	202	540	44	1877	9.29
2016–2018	217	612	51	2434	11.22
2019–2021	408	1297	80	4813	11.80
Total	1024	2945	233	9632	9.41

**Table 2:** Top 10 most-cited research articles on inclusion and disability.

Authors	Year	Title	Cites
Solish et al.	2010	Participation of children with and without disabilities in social, recreational and leisure activities	197
Hall, E.	2010	Spaces of social inclusion and belonging for people with intellectual disabilities	135
Milner & Kelly	2009	Community participation and inclusion: People with disabilities defining their place	125
McConachie et al.	2015	Systematic review of tools to measure outcomes for young children with autism spectrum disorder	121
Dingle et al.	2013	‘To be heard’: The social and mental health benefits of choir singing for disadvantaged adults	116
O’Brien et al.	2008	Exploring disability from the perspective of adults living with HIV/AIDS: Development of a conceptual framework	110
Arvanitis et al.	2009	Human factors and qualitative pedagogical evaluation of a mobile augmented reality system for science education used by learners with physical disabilities	107
Amado et al.	2013	Social inclusion and community participation of individuals with intellectual/developmental disabilities	106
Bossaert et al.	2013	Truly included? A literature study focusing on the social dimension of inclusion in education	96
Davidson, J.	2008	Autistic culture online: Virtual communication and cultural expression on the spectrum	96

**Table 3:** Top 10 most productive authors.

Authors	A	TC	TC/A	Institution	C	H Index
Bigby, C.	16	340	21.25	La Trobe University	Australia	13
Wilson, N.J.	12	158	13.17	Western Sydney University	Australia	7
O’Brien, K.K.	9	209	23.22	University of Toronto Faculty of Medicine	Canada	7
Solomon, P.	9	85	9.44	McMaster University, Faculty of Health Sciences	Canada	7
Wiesel, I.	9	164	18.22	University of Melbourne	Australia	7
Bayoumi, A.M.	8	199	24.88	University of Toronto	Canada	7
Lindsay, S.	8	163	20.38	Holland Bloorview Kids Rehabilitation Hospital	Canada	7
McConkey, R.	7	187	26.71	Ulster University	United Kingdom	4
McPherson, A.C.	7	97	13.86	University of Toronto	Canada	4
Schwab, S.	7	50	7.14	Universität Wien	Austria	4

A: number of articles published; TC: total citations; TC/A: average citations per article; C: country; H index: Hirsch index in the research line.

**Table 4:** Top 10 most productive institutions.

							TC/A	
Institution	C	A	TC	TC/A	H Index	CI (%)	CI	NCI
University of Toronto	Canada	42	617	14.69	14	19.0%	8.75	16.09
La Trobe University	Australia	25	379	15.16	12	4.0%	16.00	15.13
The University of Sydney	Australia	25	410	16.40	6	36.0%	22.33	13.06
UNSW Sydney	Australia	17	248	14.59	8	23.5%	19.25	13.15
Trinity College Dublin	Ireland	16	270	16.88	4	93.8%	17.00	15.00
Holland Bloorview Kids Rehabilitation Hospital	Canada	16	253	15.81	4	0.0%	0.00	15.81
Monash University	Australia	16	179	11.19	7	37.5%	13.83	9.60
University of Melbourne	Australia	16	283	17.69	3	37.5%	23.33	14.30
The University of British Columbia	Canada	15	68	4.53	6	26.7%	3.50	4.91
Queen’s University	Canada	13	534	41.08	4	30.8%	11.75	54.11

C: country; A: number of articles published; TC: total citations; TC/A: average citations per article; H index: Hirsch index in the research line; CI: cooperation index; TC/A CI: average number of citations with international cooperation; TC/A NIC: average number of citations without international cooperation.

**Table 5:** Top 10 most productive countries.

								TC/A	
Country	A	TC	TC/A	H Index	NC	Main Collaborators	CI	CI	NCI
United Kingdom	214	2800	13.08	53	39	Australia, Canada, United States, Germany, Ireland	22.9%	16.53	12.06
Australia	160	1947	12.17	53	34	United Kingdom, United States, Canada, The Netherlands, India	70.6%	12.51	11.34
United States	132	1449	10.98	39	28	Australia, Ireland, Canada, United Kingdom, China	39.4%	11.44	10.68
Canada	108	1514	14.02	29	14	Australia, United Kingdom, United States, Ireland, New Zealand	25.0%	8.44	15.88
Spain	82	391	4.77	29	22	Brazil, United Kingdom, United States, Colombia, Israel	25.6%	7.95	3.67
Brazil	56	210	3.75	18	6	Spain, Portugal, Canada, France, Hong Kong	16.1%	4.67	3.57
Italy	40	317	7.93	11	5	Ireland, South Korea, Spain, Turkey, United States	12.5%	2.00	8.77
South Africa	38	361	9.50	10	27	Austria, Australia, Ireland, United Kingdom, Germany	55.3%	8.14	11.18
Ireland	36	493	13.69	14	23	United States, South Africa, United Kingdom, Canada, Czech Republic.	61.1%	13.00	14.79
The Netherlands	34	594	17.47	9	18	Australia, United Kingdom, Ireland, Norway, Spain	35.3%	23.83	14.00

A: number of articles published; TC: total citations; TC/A: average citations per article; H index: Hirsch index in the research line; CI: cooperation index; TC/A CI: average number of citations with international cooperation; TC/A NIC: average number of citations without international cooperation.

**Table 6:** Top 10 most productive journals.

Journal	A	TC	TC/A	H Index Articles	H Index Journal	SJR	C
*Disability And Society*	44	707	16.07	13	76	0.85 (Q1)	United Kingdom
*Disability And Rehabilitation*	29	261	9.00	10	111	0.77 (Q1)	United Kingdom
*Journal of Applied Research in Intellectual Disabilities*	27	712	26.37	8	63	1.06 (Q1)	United Kingdom
*British Journal of Learning Disabilities*	21	148	7.05	6	39	0.63 (Q1)	United Kingdom
*International Journal of Environmental Research and Public Health*	21	57	2.71	4	113	0.75 (Q2)	Switzerland
*Journal of Intellectual Disability Research*	19	692	36.42	9	104	0.94 (Q1)	United Kingdom
*International Journal of Inclusive Education*	16	222	13.88	3	47	0.84 (Q1)	United Kingdom
*Social Inclusion*	16	93	5.81	6	17	0.51 (Q2)	Portugal
*Journal of Intellectual and Developmental Disability*	15	226	15.07	5	56	0.73 (Q1)	United Kingdom
*Tizard Learning Disability Review*	15	40	2.67	3	17	0.27 (Q3)	United Kingdom

A: number of articles published; TC: total citations; TC/A: average citations per article; SJR: SCImago Journal & Country Rank (quartile); C: country.
